# Virtual Arm Boot Camp (V-ABC): study protocol for a mixed-methods study to increase upper limb recovery after stroke with an intensive program coupled with a grasp count device

**DOI:** 10.1186/s13063-022-06047-9

**Published:** 2022-02-08

**Authors:** Lisa A. Simpson, Ruth Barclay, Mark T. Bayley, Sean P. Dukelow, Bradley J. MacIntosh, Marilyn MacKay-Lyons, Carlo Menon, W. Ben Mortenson, Tzu-Hsuan Peng, Courtney L. Pollock, Sepideh Pooyania, Robert Teasell, Chieh-ling Yang, Jennifer Yao, Janice J. Eng

**Affiliations:** 1 Graduate Program in Rehabilitation Sciences, Faculty of Medicine, University of British Columbia, Vancouver, Canada; 2 Rehabilitation Research Program, GF Strong Rehabilitation Centre, Vancouver Coastal Health, Vancouver, Canada; 3 Department of Physical Therapy, College of Rehabilitation Sciences, University of Manitoba, Winnipeg, Canada; 4 Division of Physical Medicine and Rehabilitation, University of Toronto and KITE Research Institute University Health Network, Toronto, Canada; 5 Department of Clinical Neurosciences and Hotchkiss Brain Institute, University of Calgary, Calgary, Canada; 6 Sunnybrook Health Sciences Centre, Toronto, Canada; 7 School of Physiotherapy, Dalhousie University, Halifax, Canada; 8 Department of Health Sciences and Technology, ETH, Zurich, Switzerland; 9 Department of Occupational Science and Occupational Therapy, University of British Columbia, Vancouver, Canada; 10 International Collaboration on Repair Discoveries, Vancouver, Canada; 11 Department of Physical Therapy, University of British Columbia, Vancouver, Canada; 12 Division of Physical Medicine and Rehabilitation, University of Manitoba, Winnipeg, Canada; 13 Schulich School of Medicine & Dentistry, Western University and Parkwood Institute Research, Lawson Health Research Institute, London, Canada; 14 Department of Occupational Therapy and Graduate Institute of Behavioral Sciences, College of Medicine, Chang Gung University, Taoyuan City, Taiwan; 15 Division of Physical Medicine and Rehabilitation, University of British Columbia, Vancouver, Canada; 16 University of British Columbia, 212-2177 Wesbrook Mall, Vancouver, BC V6T 1Z3 Canada

**Keywords:** Stroke, Rehabilitation, Wearable sensor, Upper extremity, Arm use, Randomized controlled trial

## Abstract

**Background:**

Encouraging upper limb use and increasing intensity of practice in rehabilitation are two important goals for optimizing upper limb recovery post stroke. Feedback from novel wearable sensors may influence practice behaviour to promote achieving these goals. A wearable sensor can potentially be used in conjunction with a virtually monitored home program for greater patient convenience, or due to restrictions that preclude in-person visits, such as COVID-19. This trial aims to (1) determine the efficacy of a virtual behaviour change program that relies on feedback from a custom wearable sensor to increase use and function of the upper limb post stroke; and (2) explore the experiences and perceptions of using a program coupled with wearable sensors to increase arm use from the perspective of people with stroke.

**Methods:**

This mixed-methods study will utilize a prospective controlled trial with random allocation to immediate or 3-week delayed entry to determine the efficacy of a 3-week behaviour change program with a nested qualitative description study. The intervention, the Virtual Arm Boot Camp (V-ABC) features feedback from a wearable device, which is intended to increase upper limb use post stroke, as well as 6 virtual sessions with a therapist. Sixty-four adults within 1-year post stroke onset will be recruited from seven rehabilitation centres. All outcomes will be collected virtually. The primary outcome measure is upper limb use measured by grasp counts over 3 days from the wearable sensor (TENZR) after the 3-week intervention. Secondary outcomes include upper limb function (Arm Capacity and Movement Test) and self-reported function (Hand Function and Strength subscale from the Stroke Impact Scale). Outcome data will be collected at baseline, post-intervention and at 2 months retention. The qualitative component will explore the experiences and acceptability of using a home program with a wearable sensor for increasing arm use from the point of view of individuals with stroke. Semi-structured interviews will be conducted with participants after they have experienced the intervention. Qualitative data will be analysed using content analysis.

**Discussion:**

This study will provide novel information regarding the efficacy and acceptability of virtually delivered programs to improve upper extremity recovery, and the use of wearable sensors to assist with behaviour change.

**Trial registration:**

ClinicalTrials.govNCT04232163. January 18, 2020.

## Background

Stroke is a leading cause of adult disability, with 40–60% resulting in upper limb hemiparesis [1]. This hemiparesis is inversely related to upper limb function, ability to use the arm and hand, and performance of activities of daily living [2].

Thousands of repetitions of reaching to grasp may be necessary to bring about upper limb functional recovery following a stroke, as suggested from animal studies [3]. Current upper limb movement practice during rehabilitation typically involves a total dose that is far below that recommended based on animal studies [4, 5]. While intensive practice early in the recovery appears to lead to better outcomes [6], high levels of practice can also result in meaningful improvements in arm function even in chronic stroke [7, 8].

Encouraging daily upper limb use and increasing intensity of practice in rehabilitation are two important goals for optimizing upper limb recovery post stroke. However, motivating thousands of repetitions in scheduled practice, or over the day is challenging. Feedback from wearable sensors may be a novel way of achieving one or both of those goals. Feedback from accelerometers have been used to increase physical activity in older adults [9] but few studies have used accelerometers to increase upper limb use. One pilot RCT used a wrist-worn accelerometer that vibrated when arm activity levels were low and showed the protocol was feasible; however, statistical analysis was not performed on the small sample (*n* = 33) [10]. Wearable sensors can be used in the home and their data accessed by the therapist. Virtual online sessions are convenient for participants who do not need to travel to a rehab centre, thereby facilitating access to intervention for those living in remote and rural communities. Virtual sessions can avoid potential transmission of infectious diseases such as COVID-19.

This mixed-methods trial has objectives to (1) determine the efficacy of a clinician directed virtual behaviour change program using an RCT where the intervention incorporates feedback from a custom wearable sensor to help increase upper limb use post stroke; and (2) explore the experiences and perceptions of using wearable sensors with a virtual program to increase arm use from the perspective of patients with stroke. This RCT uses a delayed-entry control group since we are recruiting at a point where patients are no longer receiving any formal therapy, and hence would have minimal changes without any intervention. It is hypothesized that a clinician directed virtual behaviour change program that incorporates feedback from a wearable sensor to progress the intervention will result in greater increase in arm use compared to usual care.

## Methods

**Fig. 1:**
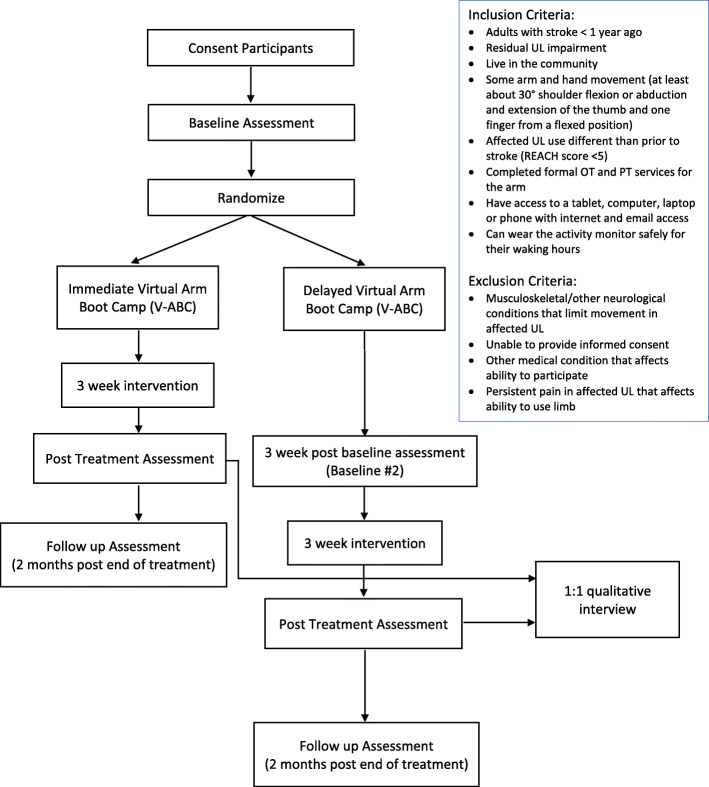
Flow diagram of mixed-methods study

This nested mixed-methods study will be comprised of a multi-centre, site-stratified, assessor blinded, parallel group, randomized controlled superiority trial with a 1:1 allocation ratio with an embedded qualitative study [11]. The control group is a wait-list group who receive the intervention after the active treatment group which facilitates recruitment and retention of participants. The design is open label with only outcome assessors being blinded so unblinding of participants will not occur. Figure 1 shows a flow diagram of the study procedures. The methods for the quantitative and qualitative components are described here separately. The intervention and evaluations will be virtual which will enable recruitment from a wider catchment, and the eventual application in local, as well as remote and rural communities.

### Quantitative component: randomized, controlled trial (RCT)

#### Setting

The RCT will recruit from 7 rehabilitation hospitals in Canada to maximize participant recruitment: GF Strong Rehabilitation Centre (Vancouver), Parkwood Institute (London), Foothills Hospital (Calgary), Riverview Health Centre (Winnipeg), Sunnybrook Health Centre (Toronto), Toronto Rehab Institute (Toronto) and Nova Scotia Rehabilitation and Arthritis Centre (Halifax). These sites are part of the Heart and Stroke Foundation Canadian Partnership for Stroke Recovery CanStroke Recovery Clinical Trials Platform. The coordinating centre will be the GF Strong Rehabilitation Centre led by Principal Investigator (Dr. Eng) and a central research team which will oversee the preparation of protocol and revisions, preparation of study documents (brochures, site training protocols, informed consent and data collection forms), publication of study reports, organization of steering committee meetings, data entry and analysis. The coordinating centre team will have access to the final data set. Individual sites will retain access to site-specific data as per contractual agreement between sites, and may request access to the final data set. The steering committee (authors of this paper) will provide agreement of the final protocol; oversee recruitment of participants at each site and liaise with study coordinator/principal investigator; review progress of study and agreeing (if necessary) on changes to the protocol to facilitate the progression and completion of study. The study team (site leads, investigators, staff coordinators) will meet at least every other week to provide trial oversight and review trial conduct, including recruitment efforts, protocol adherence and any adverse events.

#### Participant population

Adults with stroke who have been discharged or about to be discharged from the stroke programs at each rehabilitation hospital will be screened for eligibility. Individuals will be included if they (1) are within 12 months of stroke onset (with confirmed ischaemic infarct or intracerebral haemorrhage); (2) have unilateral upper limb impairment; (3) live in the community; (4) have some ability to move their arm and hand (at least about 30° shoulder flexion or abduction and extend at least one finger and thumb from a flexed position); (5) are using their affected upper limb in a different way than prior to their stroke (Rating of Arm-use in the Community and Home (REACH) Scale < 5) (12); (6) completed formal physical and occupational therapy rehabilitation for the upper limb; (7) have access to a tablet, computer, laptop or phone with internet and email access; and (8) are willing and able to wear an activity monitor safely for their waking hours. If the participant has a caregiver, they will be invited to assist in this study; however, a caregiver is not required for participating in the study unless the participant requires assistance to utilize the internet or with other aspects of the protocol.

Individuals will be excluded from the study if they (1) have musculoskeletal/other neurological conditions that limit movement in their arm; (2) are unable to provide informed consent; (3) have another medical condition that would affect their ability to participate in the treatment protocol; (4) have persistent pain in their affected upper limb that affects their ability to use the limb; and (5) are unable to speak, understand, or read English. An exception for (5) will be made if the participant does not speak the English language but has another person (i.e. family member, caretaker or friend) who can be present and translate during evaluation/treatment sessions, and home activities related to the study.

Consent will be attained by a site coordinator from each of the 7 sites. All data will be de-identified with a code prior to being transferred to the central site (University of British Columbia) where the final dataset will be stored. Initial screening will be checked from self-report over the phone. Following the screening, participants will complete an electronic consent form, facilitated by a site coordinator from each respective centre who will describe the study and answer questions about the study. Following consent, a second screening will take place with an online session to confirm the physical criteria and ensure that the videoconference software is working and the environment is appropriate for the assessment and treatment. There are no plans for collection of lab or biological specimens for genetic or molecular analysis in the current trial.

#### Randomization

Participants will be randomized after their baseline assessment at a one-to-one ratio to either the Immediate Virtual Arm Boot Camp (V-ABC) group or Delayed V-ABC group using an online, third-party, randomization service (www.randomize.net). Randomization will be in variable block sizes (to ensure allocation concealment) and stratified by site (to control for regional differences). Participants will also be stratified by the REACH score (< 3 and ≥ 3) [12]. A score of 3 separates those with mild versus moderately affected upper limbs.

#### Intervention

The intervention will be delivered by physical or occupational therapists who will be provided training over a 4-h online session. V-ABC is an intense program aimed at increasing affected upper limb movement practice delivered virtually. We define movement practice as a combination of traditional upper limb exercises (i.e. strengthening, fine motor and task-based practice) and incorporation of the affected upper limb into daily activities. The program consists of three major components: (1) Home version of the Graded Repetitive Arm Supplementary Program (H-GRASP) to increase functional capacity of the affected arm (13); (2) feedback from a wrist-worn sensor (called TENZR by Biointeractive Technologies); (3) initial treatment and follow-up treatment sessions (total 6 virtual sessions over a 3-week intervention with the first session 1.5 h, and then 1 h). The Home GRASP protocol is a self-administered exercise program that consists of range of motion, strengthening and fine motor activities [13].

##### Initial session

**Fig. 2:**
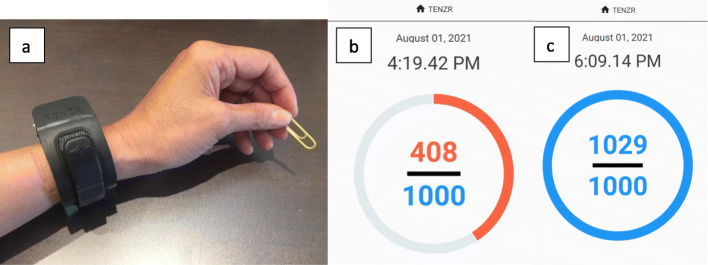
TENZR wrist-worn sensor and app. **a** TENZR wrist-worn sensor. **b** App showing a target of 1000 hand counts with 408 completed. **c** App showing a target of 1000 hand counts with 1029 completed

During the initial session, participants will be taught how to perform upper limb exercises from the Home GRASP protocol by a trained physical or occupational therapist over the Zoom software platform (Zoom Video Communications). The participants will be instructed to complete the prescribed exercises twice per day (or a minimum of 2 h/day). A binder containing verbal and pictorial instructions for each exercise and a kit with the equipment required to complete the exercises will be mailed to the participant. In addition, the TENZR and a tablet will be included in the mailed package. The TENZR does not have any visual display, and the hand counts and target (set by the therapist) are shown on an app on the tablet (see Fig. 2 of the TENZR and app). The participants will be provided with education around the importance of incorporating the affected upper limb into daily activities. The therapist will also discuss strategies for incorporating the affected upper limb into daily activities and will be provided with a checklist of example activities (e.g. open door) in which their upper limb can be used. Participants will be asked to wear the TENZR device during waking hours and to recharge the device overnight. Finally, the participants will be shown how to navigate the TENZR app on a tablet.

##### Follow-up sessions

Participants will be asked to take part in five follow-up sessions virtually after the initial session to monitor their adherence to the exercise program, ability to meet daily movement targets and to progress their exercises and movement targets. At the second session, the therapist will set daily movement targets in collaboration with the participant based on the average daily hand counts captured by the TENZR based on the baseline counts, as well as counts since the initial session. See the “Evaluations” section for the definition of a hand count. The following suggested guidelines will be provided to therapists for setting daily movement targets. If the daily baseline hand counts are less than 1000, then a target for 1000 daily hand counts will be set. If the daily baseline hand counts are greater than 1000, an additional 20% will be added. A minimum of 1000 hand counts will be set. Therapists may modify the exercises and daily activities to accommodate fatigue and/or pain.

The tablet will show the daily total GRASP counts and participants will then record these values in a 1-page summary sheet and review with their therapists at each session. Therapists will prompt participants to identify barriers to meeting movement targets and will assist participants to problem solve solutions to overcoming those barriers. The logging of the GRASP counts and discussion of strategies to increase GRASP counts will serve as a mechanism of accountability and adherence to the protocol. At the final session, the therapist will discuss recommendations for further arm activity.

The components of the V-ABC were designed to address considerations outlined in the Capability, Opportunity, Motivation, Behaviour Model applied to affected upper limb use after stroke [14]. This is a model of behaviour described in the Behaviour Change Wheel Framework [15]. The framework helps guide the development of behaviour change interventions. Table 1 outlines the Capability, Opportunity, Motivation, Behaviour Model applied to the affected upper limb use during daily activities. The information for this table was obtained from the literature and through our experience studying upper limb use post stroke.

**Table 1:** Capability, Opportunity, Motivation, Behaviour Model Components of the intervention

What needs to happen so that the affected arm is used?	Methods to address components
**Capability: Physical**	
Person needs to have enough arm function to be able to use the limb in a helpful way	• GRASP Exercise Program
**Capability: Psychological**	
• Understands the evidence related to experience-driven neuroplasticity • Understands the importance of “use it or lose it”; • Understands the importance of challenge and practice • Has emotional resilience to practice despite frustration • Has ability to problem solve when encountering problems using the limb • Has ability to monitor how and how much they are using limb • Knows how to advance task practice • Patient has sufficient memory/attention/executive function to develop and adhere to practice schedule	• Education of link between challenging practice and neuroplasticity • Feedback from TENZR • Action planning • Goal setting • Barrier identification and problem solving
**Opportunity: Physical**	
• Patient has time to use upper limb as tasks will take longer • Patient has appropriate items in the house or community to enable task practice • Patient has external reminders to use the limb	• Problem solving • Feedback from TENZR
**Opportunity: Social**	
• Has a person who will support patient while he/she is less efficient in accomplishing tasks during and after intervention • Patients not entirely driven to accomplish tasks independently no matter what • Social pressure to perform activities as before the stroke	• Social support from therapist • Social support from family
**Motivation: Reflective**	
• Believe that incorporation of their arm into daily activities will improve their function • Believe that practicing using their arm is important • Believe that temporary frustration will lead to positive gains • Be confident that their affected arm can accomplish required task • People have goals for using their limb (i.e. tasks they wish to change or start doing) • Have intentions to increase practice or use in daily activities	• Education of link between challenging practice and neuroplasticity
**Motivation: Automatic**	
• Have established routines and habits for using limb • Experience emotional benefits from using their limb	• Problem solving and planning

#### Evaluations

Participants randomized to the immediate treatment group will undergo 3 assessments through Zoom software: (1) upon admission to the study (baseline assessment), (2) immediately following completion of the treatment (post-intervention) and (3) 2 months following completion of the treatment (retention). Participants randomized to the delayed treatment group will undergo 4 assessments: (1) upon admission to the study (baseline assessment), (2) 3 weeks post baseline, (3) immediately following treatment (post-treatment) and (4) 2 months following completion of the treatment (retention). Evaluators will be occupational or physical therapists who will participate in about 4 h of training (reviewing materials and an online training session). They will be blinded to group allocation of the participant, i.e. immediate vs delayed. Participants will be instructed not to reveal their group to the evaluator. See Table 2 for the schedule of enrolment and timing of the evaluations.

**Table 2:** Schedule of enrolment and evaluations

Study procedures	Screening	Baseline evaluation	Post-intervention evaluation	Two-month evaluation
Timepoint	− *T*_1_	*T*_0_	*T*_1_	*T*_2_
Informed consent	+			
Inclusion/exclusion criteria	+			
Demographics		+		
Randomization		+		
Primary outcome measure				
3-day hand counts		+	+	+
Secondary outcome measures				
Impairment				
Upper limb pain		+	+	+
Functional				
Arm Capacity and Movement Test (upper limb functional capacity)		+	+	+
REACH Scale (self-reported arm use)		+	+	+
Stroke impact scale—arm function		+	+	+
Stroke impact scale—arm strength		+	+	+
Adverse events		+*	+*	+*

*indicates that the measure will be taken or monitored throughout the intervention period

Participants will be reminded about follow-up appointments at each appointment, in addition to a reminder immediately before the appointment by phone, text or email, depending on the participant’s preference. In addition, a secondary contact of the participant will be collected in case the participant cannot be contacted at their primary contact number.

Personal information (name, contact information) as well as demographic data (age, sex) will be collected from the participant, and medical history will be extracted from their medical chart upon consent from the participant to access their health records. Participants will be assigned a subject number that is not derived from personal information. Personal information of participants will be kept separately from the trial data and not shared between sites. No identifying information will be published or released. The data will be coded by project name and a unique alphanumeric subject number. Personal identifiers (name initials) will not be used, nor will subject number be derived from personal identifiers. Only de-identified data necessary for data analysis (demographic and assessment data) will be sent to the coordinating site, by courier or by encrypted, password-protected files. All paper data will be stored according to the university research ethics board standards (i.e. in locked cabinets within locked research offices), and electronic data will be encrypted and stored on a password-protected university server. Data entry will be carried out by a research assistant, with electronic range checks for data values, and verified by a member of the steering committee. On the consent form, participants will be informed that they can withdraw their data should they choose to withdraw from the trial.

#### Primary outcome

Study staff will document any circumstances leading to the discontinuation or modification of treatment. Regardless of whether a participant modifies or discontinues the intervention, study participants will be retained in the trial whenever possible to enable follow-up data collection of all outcome measures and prevent missing data.

Participants will be asked to wear the TENZR device on the affected upper limb for 3 days at each assessment time. Feedback from the TENZR device shown on the tablet will be inactivated during the assessment. The average daily hand counts from this 3-day assessment immediately following the 3-week intervention will be the primary outcome. This wrist-worn sensor is able to consistently capture functional reach to grasp repetitions in individuals with mild to moderate upper limb stroke impairment and has good validity, as well as good agreement over two test sessions [16]. The device uses a multi-sensor sensing approach to detect reach-to-grasp activity. Force myography is used to detect the state of the hand by monitoring the surface forces on the wrist musculo-tendonous complex proximal to the hand. The system outputs one hand count when it detects a functional reach-to-grasp movement. This is defined as any finger/grip and wrist activity which is accompanied by inertial movements of the arm before or after the hand/wrist motion [16]. For example, turning a door knob results in 1–2 hand counts; one count on reaching forward, opening the hand and grasping the door knob, and a second count can occur if there is a distinct hand opening on letting go of the door knob and withdraw of the arm. The combination of an arm reaching movement and a hand grasping/releasing movement approximates the initial interaction with an object (i.e. reach and grasp) and/or final interaction with an object (i.e. release and arm move away).

#### Secondary outcomes

##### Self-report affected arm use

The REACH Scale is a valid and reliable tool for capturing how people are using their affected arm and hand outside in the community setting [12].

##### Upper limb functional capacity

The Arm Capacity and Movement Test was developed by our research team to capture participants’ ability to use their affected upper limb virtually and has been shown to be valid and reliable [17].

##### Upper limb pain

A 0–10 visual analogue pain scale will be used to capture upper limb pain throughout the treatment and at each assessment time point. This measure has been shown to be valid and reliable across many different populations [18].

##### Stroke specific health quality of life (strength and arm function subscales)

Two subscales of the *Stroke Impact Scale* (SIS) will be used to capture the impact of stroke on participants’ strength and arm function. The SIS has excellent measurement properties for people with stroke [19].

#### Data monitoring

All sites will report minor and serious adverse events that occur from baseline through to the 2-month follow-up. An internal Data Safety Monitoring Board comprised of 5 physical medicine physicians will be notified of adverse events when they arise. An internal board is deemed to match the low risk of this trial. No interim analyses will be done and there will be no stopping rule for the trial because it is not anticipated that any serious adverse events would result from the protocol. Protocol modifications will be communicated to investigators and trial staff via email and discussed at the weekly meetings, and documentation will be updated as appropriate (e.g. updated protocol, amendments and approval by the ethics board, update of the clinical trial registry protocol). Participants will be advised of protocol changes and invited to sign an amended consent form to indicate their continued consent to participate in the study.

#### Sample size estimates

The sample size was calculated using G power. The study will require 32 patients per group to achieve 80% power at a level of significance of 0.05 to detect a large effect size (Cohen’s *f* = 0.4) using an ANCOVA model (controlling for baseline values) and a dropout rate of 20%.

#### Data analyses

Descriptive statistics will be used to summarize data. Analysis of covariance will be used for the main analysis to estimate the differences comparing the 3-day hand counts at the post-treatment (immediately following the 3-week intervention) controlling for the baseline. Multiple imputation will account for missing data. The significance level will be set at 0.05, and all statistical tests will be two-tailed. Participant data will be analysed on an intention-to-treat basis. Confidence intervals (95% CIs) will be reported, where applicable. Secondary outcomes at post-treatment will follow the same analysis. In an additional analysis, we will use a mixed effects model to examine whether the intervention effects are retained at 2 months after the intervention ends.

### Qualitative component: qualitative description

A qualitative descriptive study will be conducted alongside with the RCT to explore patients’ views and experiences of the virtual ABC program, including the TENZR activity monitor, GRASP exercises, therapy sessions and trying to use their affected arms more during daily activities. The qualitative description methodology is widely used for research questions focusing on understanding patients’ experiences, particularly for examining healthcare phenomena [20]. It is a rigorous methodology that provides a comprehensive and straight description of an experience without the need to apply a certain theory or framework [21]. The consolidated criteria for reporting qualitative research guidelines was used to inform the qualitative design and will be used to report the qualitative data to ensure methodological quality and transparent reporting [22].

#### Participants

Participants who complete the virtual ABC program will be recruited from all sites to participate in qualitative interviews provided they are able to communicate fully (English proficiency, non-severe aphasia). It is expected that 15–20 participants will be eligible and interviewed virtually and will depend on when data sufficiency is reached, and identified themes do not need to be adjusted by further data collected [23].

#### Procedures / Data collection

Individual semi-structured interviews will be conducted online using Zoom software. Interviews will be conducted within 2 months of participants completing the V-ABC program. Interviews will be conducted by a trained researcher with qualitative experience, who is not involved in the care of the participant. Interviews will last approximately 60 min and will be audio-recorded.

The semi-structured interview guide was developed by one of the authors (CL Yang) and reviewed by two physical therapists, two occupational therapists, two individuals with stroke and an engineer who was involved in the development of the TENZR activity monitor (Appendix 1). The development of the interview guides was informed by the Capability, Opportunity, Motivation, Behaviour Model to understand the mechanisms of changes within a behaviour change intervention [15]. This model positions behaviour as the product of an interaction between three components: capability, opportunity and motivation [15]. Questions aim to identify relevant components of the virtual ABC program which contribute to its effectiveness.

#### Data processing and analysis

All interviews will be transcribed verbatim and analysed using conventional content analysis [24]. Transcripts will be read and re-read to ensure familiarization with the data. Statements will be inductively coded to initial codes that encompass the meaning of the statement by two investigators. The initial codes will then be iteratively consolidated and grouped into broad categories, which will then be used to develop common themes. Through an iterative process, themes and interpretations will be refined to provide an understanding of how the virtual ABC program is perceived by the participants.

#### Trustworthiness

Several strategies will be used to ensure the trustworthiness of this qualitative component of the study. Investigator triangulation will be applied by involving several researchers in the process of data analysis to support the credibility of the qualitative findings. Regular analytical sessions will be held within the research team to compare interpretations and resolve the differences. Negative case analysis and participant checking will also be used to enhance credibility. The synthesized data will be presented to the participants to check for accuracy and resonance with their experiences. If any additional data were to be added during this process, it will be cross-referenced with existing codes and integrated into the analysis [25]. Furthermore, interpretation of the qualitative data alongside the quantitative findings will also enhance the credibility of this study. Research reflexivity will be facilitated by keeping a reflexive journal to support the transferability of the qualitative findings. Reflections on any assumptions, power differentials (e.g. between participant and interviewer) and interpersonal dynamics that come up during interviews that may affect data collection will be noted to account for the positions of the interviewers and personal assumptions [26]. In addition, information regarding research context, processes, participants, context and researcher-participant relationships will be provided to allow the reader to evaluate the rigour and transferability of the study.

#### Knowledge translation

The data collected in this study will be used for journal publications and presentations to clinicians, researchers and the public. Participants will receive trial outcome information if requested.

## Discussion

The integration of wearable sensors with rehabilitation programs is in its infancy. There have been some programs which utilize heart rate monitors and step counters to set targets to improve walking or physical activity after stroke [27, 28]. However, the use of monitors for the upper limb treatment after stroke are less common. Sanders et al. [29] used a grip sensor integrated with a musical computer game (MusicGlove) to encourage hand use after stroke. Da-Silva et al. [10] showed preliminary results on using a wrist-worn accelerometer which vibrated when arm activity levels were low. The integration of a sensor to monitor upper limb use may be ideal for home programs. When home programs are prescribed during rehabilitation, it is difficult for the therapist to determine how much practice is actually occurring. While a patient can document the minutes of practice, one person could practice 30 min with a few hundred repetitions, while another could do a thousand repetitions. Additionally, the use of a wearable sensor can provide valuable objective data with respect to incorporation of the affected upper limb into ADLs throughout the day, a critical piece to optimizing functional gains. As the Capability, Opportunity, Motivation, Behaviour Model illustrates [14, 15], changing behaviour is complex. In our study, feedback from the sensor can provide accountability, as well as motivation for the number of repetitions that the patient is undertaking in their home program and in daily use. The proposed protocol is innovative in focusing on use as the primary outcome. Ultimately, the goal of rehabilitation is to improve use of their arm in everyday life, and not just in the clinic.

There are many reasons why the development of evidence-based virtual rehabilitation programs is needed. The COVID-19 pandemic has highlighted the benefits of virtual care as hospital programs experienced restricted caseloads due to the risk of on-site COVID-19 transmission. A program like V-ABC can be delivered at home without risk to COVID-19. Many rehabilitation programs quarantined patients to their room for a period of time to reduce the potential for infection. Social distancing requirements reduced the space available to treat patients, and notably, permitted caseloads were reduced. We previously showed the feasibility of using a virtual arm and hand rehabilitation protocol in a small pretest-posttest study to improve arm function without any specialized equipment or technology except for the communication device itself (computer, tablet, smartphone) [30]. Beyond COVID-19, evidence-based virtual programs have appeal. They increase the reach of a program, especially to rural and remote locations. It has been documented that people living in rural regions have less chance of receiving physical therapy or occupational therapy after a stroke [31]. Treatment in the home also provides a convenience to the patient. Lastly, there may be cost-savings in having a virtual program at the home of a patient. The patient does not need to travel to the hospital, nor does the therapist need to travel to provide home therapy, and caseloads are not restricted by space.

This study uses a mixed-methods approach to study the efficacy and acceptability of this program. It is important to understand the experience of the patient in utilizing a wrist-worn sensor, in conjunction with an exercise program. Understanding the barriers and facilitators to the protocol will facilitate implementation if the protocol demonstrates efficacy.

This study has several limitations. We anticipate individuals with mild to moderate stroke severity to be eligible, while those with severe stroke who have little hand or arm movement would not be eligible.

## Trial status

Protocol version 8, last amendment to protocol on Sept 28, 2020. Recruitment commenced September 9, 2020, with recruitment expected to complete September 2022. Approximately 50% of participants are recruited at this time.

## Data Availability

The datasets to be used in the proposed study will be available 12 months after the publication of the trial at UBC Dataverse, a publicly accessible data repository.

## Appendix 1. Stroke Participant Interview Guide

Introduction: Today I want to ask you some questions about what it was like taking part in the Arm Boot Camp 3-week program. You might have already answered a survey about the Arm Boot Camp program. The purpose of this interview is to understand more about your thoughts on the Arm Boot Camp program, which includes the activity monitor (the one you wore during the program), GRASP exercises (i.e. arm and hand exercises in the manual that we provided to you), therapy sessions, and trying to use of your affected arm more during daily activities. You also took part in the evaluation sessions. As the evaluation sessions are to assess the effects of the Arm Boot Camp program, not parts of the training program, we will not include the evaluation sessions in our discussion today.

**Start recording.**

1. General: age, sex, time post stroke, hemisphere affected, dominant side prior and post stroke, education, work-status, living, marital status
2. Why were you interested in taking part in the Arm Boot Camp 3-week program?
3. How did you feel about your affected arm and hand function before you started the Arm Boot Camp program? Prompt: Range of motion, strength, coordination in your affected arm and hand, etc.
4. How do you feel about your affected arm and hand function now?
5. What did you think about the Arm Book Camp 3-week program?
  Prompt: What, if anything, did you like about the program (e.g. activity monitor, GRASP exercises, therapy sessions, trying to use your affected arm more during daily activities)?
  Prompt: How, if at all, could it be improved?
6. What was your experience with the activity monitor (show the participant the TENZR activity monitor)?
  Prompt: What are your thoughts on knowing how much your affected hand move over a day?
  Prompt: How have you progressed on the activity monitor? (or How have the targets been progressed over time?)
  Prompt: What, if anything, did you like about the activity monitor (e.g. feedback, set goals)?
  Prompt: How, if at all, could it be improved (e.g. ease of use, comfort)?
  Prompt: What are your thoughts on using the activity monitor beyond this trial for yourself?
7. What was your experience with the GRASP exercises?
  Prompt: How have you progressed on the GRASP exercises?
  Prompt: During the Arm Boot Camp 3-week program, you were asked to do the GRASP exercises twice a day or at least 2 hours a day. What was your experience trying to do this amount of GRASP exercise program?
  Prompt: What, if anything, worked well with the exercises (e.g. equipment, exercises, log sheets)?
  Prompt: How, if at all, could it be improved?
  Prompt: What are your thoughts on using the GRASP exercises beyond this trial for yourself?
8. What was your experience with the therapy sessions (what was your experience of the sessions with your therapist)?
  Prompt: What was the content of the therapy sessions (e.g. education, support, exercise progression, setting goals, motivation, encouragement, etc.)?
  Prompt: What, if anything, worked well with the therapy sessions?
  Prompt: How, if at all, could it be improved?
9. What was your experience with trying to use your affected arm and hand more in daily activities during the Arm Boot Camp 3-week program?
  Prompt: How was incorporating your affected arm and hand in daily activities discussed during the therapy sessions? What daily activities, if anything, did you do to increase the hand counts?
  Prompt: During the 3-week program, the activity monitor showed the number of hand counts over the course of an average day. To what extent, if any, do you think your hand counts were due to the GRASP exercises?
  Prompt: To what extent, if any, do you think your hand counts were due to using your affected arm during the rest of the day (during daily activities outside of GRASP exercises)?
  Prompt: What did you think about the idea that using your affected arm during daily activities is therapy?
10. The therapy sessions were delivered virtually using videoconferencing technology (e.g. Zoom, webcam, computer, headphones, etc.). What was your experience with the virtual aspect of the sessions?
  Prompt: What had been your experience with using videoconferencing for therapy sessions before the study?
  Prompt: How do you feel about using videoconferencing for therapy sessions now?
  Prompt: What, if anything, worked well with the virtual sessions?
  Prompt: How, if at all, could it be improved?
  Prompt: What are your thoughts on using videoconferencing technology for therapy sessions beyond this trial for yourself?
11. What do you think about your family or caregiver’s involvement in the Arm Boot Camp program?
  Prompt: What was your family or caregiver’s role in this program?
  Prompt: What, if anything, worked well with their involvement?
  Prompt: How, if at all, could it be improved?
12. What was your experience trying to continue doing exercises with your affected arm after the Arm Boot Camp 3-week program was over?
  Prompt: What, if any, exercises do you do now with your affected arm? (If they say they are doing exercises now for their affected arms, clarify if the exercises are the GRASP exercises or include some of the GRASP exercises.)
  Prompt: How much exercise do you do now with your affected arm?
  Prompt: How much do you now use your affected arm during daily activities?
  Prompt: What, if any, were some of the barriers to doing exercises for your affected arm after the program was over?
  Prompt: What, if anything, helped you continue doing exercises for your affected arm after the program was over? (if appropriate)
13. Is there anything that you would like to say but did not get a chance to say?

## Abbreviations

V-ABC: Virtual Arm Boot Camp
REACH: Rating of Arm-use in the Community and Home Scale
Home GRASP: Home version of the Graded Repetitive Arm Supplementary Program
