# Correction: Virtual Arm Boot Camp (V-ABC): study protocol for a mixed-methods study to increase upper limb recovery after stroke with an intensive program coupled with a grasp count device

**DOI:** 10.1186/s13063-022-06134-x

**Published:** 2022-03-11

**Authors:** Lisa A. Simpson, Ruth Barclay, Mark T. Bayley, Sean P. Dukelow, Bradley J. MacIntosh, Marilyn MacKay-Lyons, Carlo Menon, W. Ben Mortenson, Tzu-Hsuan Peng, Courtney L. Pollock, Sepideh Pooyania, Robert Teasell, Chieh-ling Yang, Jennifer Yao, Janice J. Eng

**Affiliations:** 1 Graduate Program in Rehabilitation Sciences, Faculty of Medicine, University of British Columbia, Vancouver, Canada; 2 Rehabilitation Research Program, GF Strong Rehabilitation Centre, Vancouver Coastal Health, Vancouver, Canada; 3 Department of Physical Therapy, College of Rehabilitation Sciences, University of Manitoba, Winnipeg, Canada; 4 Division of Physical Medicine and Rehabilitation, University of Toronto and KITE Research Institute University Health Network, Toronto, Canada; 5 Department of Clinical Neurosciences and Hotchkiss Brain Institute, University of Calgary, Calgary, Canada; 6 Sunnybrook Health Sciences Centre, Toronto, Canada; 7 School of Physiotherapy, Dalhousie University, Halifax, Canada; 8 Department of Health Sciences and Technology, ETH, Zurich, Switzerland; 9 Department of Occupational Science and Occupational Therapy, University of British Columbia, Vancouver, Canada; 10 International Collaboration on Repair Discoveries, Vancouver, Canada; 11 Department of Physical Therapy, University of British Columbia, Vancouver, Canada; 12 Division of Physical Medicine and Rehabilitation, University of Manitoba, Winnipeg, Canada; 13 Schulich School of Medicine & Dentistry, Western University and Parkwood Institute Research, Lawson Health Research Institute, London, Canada; 14 Department of Occupational Therapy and Graduate Institute of Behavioral Sciences, College of Medicine, Chang Gung University, Taoyuan City, Taiwan; 15 Division of Physical Medicine and Rehabilitation, University of British Columbia, Vancouver, Canada; 16 University of British Columbia, 212-2177 Wesbrook Mall, Vancouver, BC V6T 1Z3 Canada

**Correction to: Trials 23, 129 (2022)**

**https://doi.org/10.1186/s13063-022-06047-9**

Following the publication of the original article [1], we were notified that one of the author names has been incorrectly spelled.

Originally published name: McKay-Lyons M.

Corrected name: MacKay-Lyons M.

The original article has been corrected.

## References

[1] Simpson (2022). Virtual Arm Boot Camp (V-ABC): study protocol for a mixed-methods study to increase upper limb recovery after stroke with an intensive program coupled with a grasp count device. Trials.

